# Association of Eating Alone With Depression Among Older Adults Living Alone: Role of Poor Social Networks

**DOI:** 10.2188/jea.JE20190217

**Published:** 2021-04-05

**Authors:** Ryota Sakurai, Hisashi Kawai, Hiroyuki Suzuki, Hunkyung Kim, Yutaka Watanabe, Hirohiko Hirano, Kazushige Ihara, Shuichi Obuchi, Yoshinori Fujiwara

**Affiliations:** 1 Research Team for Social Participation and Community Health, Tokyo Metropolitan Institute of Gerontology, Tokyo, Japan; 2 Research Team for Human Care, Tokyo Metropolitan Institute of Gerontology, Tokyo, Japan; 3 Research Team for Promoting Independence and Mental Health, Tokyo Metropolitan Institute of Gerontology, Tokyo, Japan; 4 Gerodontology, Department of Oral Health Science, Faculty of Dental Medicine, Hokkaido University, Sapporo, Japan; 5 Department of Social Medicine, Hirosaki University Graduate School of Medicine, Aomori, Japan

**Keywords:** eating alone, social network, living alone, depression, older adults

## Abstract

**Objectives:**

Eating alone is associated with an increased risk of depression symptoms. This association may be confounded by poor social networks. The present study aimed to determine the role of poor social networks in the association of eating alone with depression symptoms, focusing on cohabitation status.

**Methods:**

Seven hundred and ten community-dwelling older adults were categorized according to their eating style and social network size, evaluated using an abbreviated version of the Lubben Social Network Scale, with poor social network size (defined as the lowest quartile). Living arrangements and depression symptoms, detected using the Zung Self-Rating Depression Scale, were also assessed.

**Results:**

A mixed-design two-way analysis of covariance (eating style and social network size factors) for the depression scale score, adjusted by covariates, yielded significant effects of social network size and eating style without interaction. Greater depression scores were observed in eating alone and poor social network size. Analysis of participants living with others showed the same results. However, among older adults living alone, only a significant main effect of social network size was observed; poor social network size resulted in greater depression scores irrespective of eating style.

**Conclusions:**

Poor social network size, and not eating alone, was associated with greater depression symptoms among older adults living alone, whereas both factors may increase depression symptoms among older adults living with others. Poor social network size may show a stronger influence on depression than eating alone in older adults living alone; thus, social network size is an important health indicator.

## INTRODUCTION

There has been growing interest in the health impacts of eating alone among older adults. Some studies show that this eating style may be linked to problems, including depression symptoms,^1^^–^^3^ obesity,^4^ metabolic syndrome,^5^ and increased risk of mortality.^6^ All these health outcomes caused by eating alone seem to be prevalent among older adults living alone.

However, the adverse health effect of eating alone may be confounded by poor social network size.^7^ A recent longitudinal study has demonstrated that the influences of a poor social network on health may outweigh the influences of living alone.^8^ Given that eating alone is influenced by living arrangements but is essentially a consequence of social isolation (ie, poor social network), the social network size of at-risk populations may be the better factor to focus on to improve public health.

The aim of the present study was to determine whether the association between eating alone and depression symptoms, which is an accepted adverse health outcome caused by eating alone in older adults who live alone, can be attributed to a poor social network. Following previous findings, we hypothesize that, among older adults living alone, poor social networks are more strongly associated with greater depression symptoms than eating alone. Our findings would extend and improve the previous findings regarding the combined effects of eating alone and living alone with depression symptoms.

## METHODS

### Study design and participants

This was a cross-sectional analysis of an ongoing prospective cohort study named the ‘Otassya study’, which was conducted in Itabashi, an urban ward in north-west Tokyo. The study aimed to determine the health condition among older adults living in the community. The design and logistics of the study has been described in detail elsewhere.^9^^,^^10^ We analyzed the data collected in 2017, which introduced a questionnaire on eating style and social network size. Among 1,564 older adults who intended to participate in the cohort study and received recruitment letters, 761 participated in the comprehensive health check-up held in 2017. Exclusion criteria for the analysis were severe cognitive decline (Mini-Mental State Examination score <24), which cast doubt on the reliability of participants’ responses (*n* = 16) and incomplete data (*n* = 35). As a result, 710 participants were included. No participants reported problems with their activities or instrumental activities of daily living.

Ethics approval was obtained from the Tokyo Metropolitan Institute of Gerontology Ethics Board for the research protocol (No. 28-2017). Participants’ signed informed consent was obtained during enrolment, before study assessments.

### Measurements

#### Eating style, social network size, and living arrangement

Participants were asked: ‘Do you eat a meal with others at least once a day?’ Those who answered ‘no’ were assigned to the eating alone group, while those who answered ‘yes’ were assigned to the not eating alone group.^2^

We applied an abbreviated version of the Lubben Social Network Scale (LSNS-6) to evaluate size of social networks. The LSNS-6 includes six items and can assess the size of active and intimate networks of family ties and friendship ties with whom participants could talk to or call for help.^11^

#### Depression symptoms and covariates

We assessed participants’ depression symptoms using the Japanese version of the Zung Self-Rating Depression Scale (SDS).^12^ The SDS consists of 20 questions, with total scores ranging from 20 to 80. Higher scores indicate greater depression symptoms.^13^ We converted these raw scores into index scores (25–100).^14^

Demographic characteristics of participants, including gender, age, education level, subjective health, subjective financial situation, and numbers of comorbidities and medications, were assessed and recorded as possible covariates of the associations of depression symptoms with eating alone and poor social networks, based on previous findings.^15^^–^^17^ Subjective financial situation was assessed based on a previously validated questionnaire, using a five-point scale that classified participants as either having a moderate or a poor financial situation.^8^

### Data analysis

To define poor social networks, the LSNS-6 scores were divided into quartiles, with the lowest (fourth) quartile considered as the poor social network group.^8^ We then divided the participants into four groups: i) not eating alone with decent social network, ii) not eating alone with poor social network, iii) eating alone with decent social network, and iv) eating alone with poor social network.

The participants’ characteristics were summarized and the differences between the four groups were analyzed using chi-square tests and analysis of variance (ANOVA), as appropriate. For the SDS score, two-way analyses of covariance (ANCOVA) with two independent factors, eating style (not eating alone or eating alone) and size of social network (poor or decent social network), were performed separately for all participants (ie, participants living with others and participants living alone). The ANCOVA included gender, age, education level, subjective health, subjective financial situation, and numbers of medications and comorbidities as covariates.

Statistical significance was set at an alpha level of 0.05. All statistics were performed using the Statistical Package for the Social Sciences version 23.0 (SPSS, SPSS Inc., Chicago, IL, USA).

## RESULTS

Table 1 shows participant’s characteristics stratified by eating style and social network size. There were 197 participants who ate alone (27.7% of the total sample) and 174 participants with poor social networks (24.5% of the total sample). The chi-squared tests and ANOVAs revealed that the proportion of participants with five or more medications, diabetes, poor subjective health, and poor subjective financial situation tended to be higher in groups with poor social networks. Further, the proportion of female participants was highest in the group that ate alone but had a decent social network and lowest in the group that ate with others but had a poor social network. Those who ate alone naturally showed significantly higher proportion of living alone.

**Table 1.** Participant characteristics stratified by the combinations of eating style and social network size

	Not EA and decent SN	Not EA and poor SN	EA and decent SN	EA and poor SN	*P*-value

	*n* = 411	*n* = 102	*n* = 125	*n* = 72	
	(57.9%)	(14.4%)	(17.6%)	(10.1%)	
Female, *n* (%)	234 (56.9)	38 (37.3)^*^	96 (76.8)^*^	47 (65.3)	<0.001
Age, mean (SD)	73.2 (6.0)	74.4 (6.7)	75.7 (6.3)^†^	74.0 (6.8)	0.001
Years of education, mean (SD)	13.5 (2.7)	13.0 (3.1)	13.0 (2.3)	12.5 (2.4)^†^	0.015
Body mass index, mean (SD)	22.9 (3.4)	22.6 (3.1)	22.5 (3.4)	22.9 (3.7)	0.697
Number of comorbidities, mean (SD)	1.29 (1.15)	1.37 (1.22)	1.34 (1.02)	1.65 (1.18)	0.104
Five plus medications, *n* (%)	90 (21.9)^*^	32 (31.4)	35 (28.0)	33 (45.8)^*^	<0.001
Hypertension, *n* (%)	172 (41.8)	40 (39.2)	50 (40.0)	41 (56.9)	0.073
Heart disease, *n* (%)	63 (15.3)	16 (15.7)	20 (16.0)	16 (22.2)	0.536
Diabetes mellitus, *n* (%)	41 (10.0)^*^	18 (17.6)	14 (11.2)	15 (20.8)^*^	0.021
Osteoarthritis, *n* (%)	62 (15.1)	21 (20.6)	19 (15.2)	11 (15.3)	0.584
Cancer, *n* (%)	64 (15.6)	21 (20.6)	19 (15.2)	9 (12.5)	0.498
Poor subjective health, *n* (%)	46 (11.2)^*^	18 (17.6)	15 (12.0)	16 (22.2)^*^	0.039
Poor subjective financial status, *n* (%)	69 (16.8)^*^	30 (29.4)^*^	16 (12.8)^*^	21 (29.2)^*^	0.001
Living alone, *n* (%)	30 (7.3)^*^	5 (4.9)	91 (72.8)^*^	56 (77.8)^*^	<0.001
Drinking habit, *n* (%)	221 (53.8)^*^	56 (54.9)	46 (36.8)^*^	33 (45.8)	0.006
Smoking habit, *n* (%)	32 (7.8)	7 (6.9)	14 (11.2)	8 (11.1)	0.495
MMSE, mean (SD)	28.7 (1.8)	28.1 (2.6)	28.3 (2.2)	28.4 (1.6)	0.035
LSNS-6, mean (SD)	18.4 (4.3)	7.7 (2.7)^†‡^	17.7 (4.4)	6.9 (3.3)^†‡^	<0.001

EA, eating alone; LSNS-6, Lubben Social Network Scale; MMSE, Mini-Mental State Examination; SN, social network.

^*^Significant adjusted residuals; ^†^significant difference with Not EA and decent SN (*P* < 0.0125, Bonferroni correction); ^‡^significant difference with EA and decent SN (*P* < 0.0125, Bonferroni correction).

**Figure 1.**
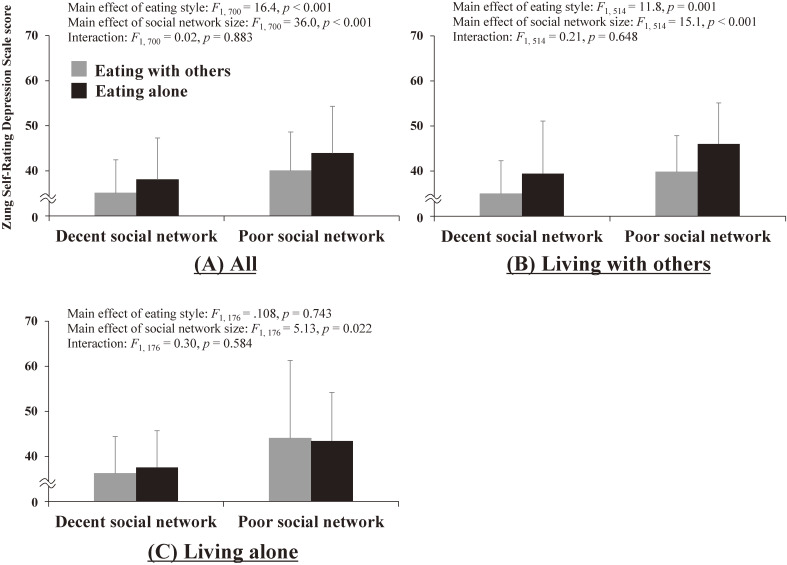
Zung Self-Rating Depression Scale score comparisons by eating style and social network size among (A) all participants, (B) participants living with others, and (C) participants living alone. Each analysis of covariance was adjusted for gender, age, education level, subjective health, subjective financial situation, and numbers of medications and comorbidities.

Figure 1 shows the results of the two-way ANCOVA for SDS score depending on eating style and social network size among all participants: those who live with others and those who live alone. For all participants, the results showed significant main effects of both eating style and social network size, and no significant interaction between the two factors. This indicates that both eating alone and poor social network size result in greater SDS scores. For the stratified analyses, those living with others showed the same results as the analysis for all participants. However, those living alone showed a significant main effect only with social network size (ie, not with eating alone), indicating that poor social network size results in greater SDS scores irrespective of eating style.

## DISCUSSION

The results demonstrated that both eating alone and poor social network among older adults who lived with others were associated with greater depression symptoms, irrespective of the presence of one or the other. However, in support of our hypothesis, poor social network size resulted in greater SDS scores irrespective of eating style in older adults living alone. Our results are aligned with the recent finding that, among older adults, living alone is not a factor of social vulnerability of their health condition, and that instead, poor social network should be considered a social aspect of health risk factors among older aged individuals.^8^ Our findings further suggest that the association between eating alone and poor mental health presented by depression symptoms in older adults who live alone is entangled with poor social network, so eating alone may not be a simplex risk factor for mental illness, including depression. This study contributes to correcting claims that eating alone is a harmful lifestyle among every older adult living alone.

Although caution should be exercised when interpreting a cross-sectional design, the findings of the present study suggest that the influence of poor social networks on the mental health of older adults living alone may outweigh the influences of eating alone. Humans are intrinsically social beings for whom social ties significantly impact overall health and well-being. Given this, it is not surprising that social isolation is a well-known determinant of health deterioration.^18^^–^^22^ Therefore, insofar as eating alone is deemed a risk factor for mental illness, this is probably ultimately based on social isolation.

On the other hand, both eating alone and poor social network independently resulted in greater depression symptoms among older adults who lived with others. This is partially consistent with previous findings that older adults who ate meals alone but lived with others had an elevated risk of depression symptoms and mortality.^2^^,^^6^ Eating alone among older adults who live with others is an unusual lifestyle, implying domestic social isolation (eg, neglect). The main effect of eating style on SDS seen in older adults who lived with others is likely a result of this unusual situation that is ultimately destructive to mental health, independent of social network size.

Our results are partly inconsistent with previous findings in which significant association between eating alone and depression was independent of social factors, including types of social support (eg, relative or friend and neighbor), belonging to a social club (eg, volunteer group and sports groups), and frequency of meeting friends. The reason for this inconsistency may be due to the methodological approach to assessing the degree and types of social ties. The previous study assessed social ties using three single questions as mentioned above, whereas LSNS-6 used in our study specializes in evaluating social network size with scoring. Detailed examination and assignment of poor social network in the present study probably yielded the difference from a previous finding. Further longitudinal studies will be needed to examine our speculation for the inconsistency between previous and present findings, focusing on degree and types of social ties.

Our study improves on the prior findings by adding new and significant results; however, the small number of men, which precludes our ability to perform stratified analyses for older adults who were living alone and living with others based on gender, is a limitation. A previous study indicated that the influence of eating alone on depression symptoms was observed in older men who were living alone.^1^ Although we introduced gender into each ANCOVA as a covariate and found no significant effect (data not shown), the gender difference for the findings of this study needs to be examined in future studies.

### Conclusions

A notable point from the findings is that, even when eating alone and living alone, older adults who keep decent social networks are more likely to show good mental health. This work suggests that health care providers should also focus on poor social networks as a hazardous factor that may have a negative impact on mental health among older adults who are living alone, rather than just on eating alone.
